# Inter- and intraobserver reliability of non-weight-bearing foot radiographs compared with CT in Lisfranc injuries

**DOI:** 10.1007/s00402-020-03391-w

**Published:** 2020-03-05

**Authors:** Ville T. Ponkilainen, Nikke Partio, Essi E. Salonen, Antti Riuttanen, Emma- Liisa Luoma, Gilber Kask, Heikki-Jussi Laine, Heikki Mäenpää, Outi Päiväniemi, Ville M. Mattila, Heidi H. Haapasalo

**Affiliations:** 1 School of Medicine, University of Tampere, 33520 Tampere, Finland; 2 Department of Orthopaedics and Traumatology, Faculty of Medicine and Life Sciences and Tampere University Hospital, University of Tampere, Teiskontie 35, PL2000, 33521 Tampere, Finland; 3 COXA Hospital for Joint Replacement, Biokatu 6, 33520 Tampere, Finland

**Keywords:** Lisfranc, Injury, Radiographs, X-ray, Interobserver, Intraobserver, Reliability, Responsiveness

## Abstract

**Background:**

Injury of the tarsometatarsal (TMT) joint complex, known as Lisfranc injury, covers a wide range of injuries from subtle ligamentous injuries to severely displaced crush injuries. Although it is known that these injuries are commonly missed, the literature on the accuracy of the diagnostics is limited. The diagnostic accuracy of non-weight-bearing radiography (inter- or intraobserver reliability), however, has not previously been assessed among patients with Lisfranc injury.

**Methods:**

One hundred sets of foot radiographs acquired due to acute foot injury were collected and anonymised. The diagnosis of these patients was confirmed with a CT scan. In one-third of the radiographs, there was no Lisfranc injury; in one-third, a nondisplaced (< 2 mm) injury; and in one-third, a displaced injury. The radiographs were assessed independently by three senior orthopaedic surgeons and three orthopaedic surgery residents.

**Results:**

Fleiss kappa (κ) coefficient for interobserver reliability resulted in moderate correlation κ = 0.50 (95% CI: 0.45– 0.55) (first evaluation) and κ = 0.58 (95% CI: 0.52–0.63) (second evaluation). After three months, the evaluation was repeated and the Cohen’s kappa (κ) coefficient for intraobserver reliability showed substantial correlation κ = 0.71 (from 0.64 to 0.85). The mean (range) sensitivity was 76.1% (60.6–92.4) and specificity was 85.3% (52.9–100). The sensitivity of subtle injuries was lower than severe injuries (65.4% vs 87.1%* p* = 0.003).

**Conclusions:**

Diagnosis of Lisfranc injury based on non-weight-bearing radiographs has moderate agreement between observers and substantial agreement between the same observer in different moments. A substantial number (24%) of injuries are missed if only non-weight-bearing radiographs are used. Nondisplaced injuries were more commonly missed than displaced injuries, and therefore, special caution should be used when the clinical signs are subtle.

**Level of evidence:**

III.

## Introduction

Lisfranc injury was originally described as a partial or complete dislocation of the tarsometatarsal (TMT) joints, although the definition and classifications of the injury have altered over the years [39, 45]. Indeed, multiple classifications have been presented, yet there is still no consensus on the precise definition of Lisfranc injuries [6, 25, 33]. Nevertheless, Lisfranc injury is recognized nowadays as a wide variety of both bony and ligamentous injuries of the TMT joint region ranging from subtle ligamentous injuries to severely displaced or crush injuries [21, 25, 33, 35, 43, 45].

The incidence of Lisfranc injuries has been presented to be 9.2/100 000 person-years when diagnosed with computed tomography (CT) [34]. Furthermore, it has been estimated that even Lisfranc injuries resulting from high-energy trauma mechanisms can be initially overlooked or misdiagnosed in 20%–24% of cases [17, 30]. However, the current literature on the accuracy of the diagnostics is limited. Primary diagnosis is usually based on non-weight-bearing radiographic imaging, though its sensitivity has been estimated to be quite low (24%–50%) when compared with CT [17, 38] Weight-bearing radiographs or magnetic resonance imaging (MRI) are suggested modalities for detecting ligamentous injuries [33, 36–38], yet it may be impossible to acquire weight-bearing images due to the extensively painful foot at the first presentation [33, 36–38, 40, 53]. In their systematic review, Sripanich and colleagues [50] reported that CT scans seem to be currently the most precise imaging modality in detecting bony injuries; whereas, MRI seems to be the most precise in detecting ligamentous injuries. It has also been reported that the sensitivity of the weightbearing radiograph is not higher compared with the non-weight-bearing radiograph and is less sensitive than CT [38]. Nevertheless, many of the previously published studies have still relied on non-weight-bearing or weight-bearing radiographs [8, 9, 12, 20, 23, 29, 33, 35, 41, 47].

When evaluating the accuracy of the diagnosis, it is important to evaluate the reliability (interobserver reliability) and the reproducibility (intraobserver reliability) of the diagnostic test [22]. The interobserver reliability is a method to evaluate the correlations between the observers as mathematical measures [5, 19]. The intraobserver reliability, in turn, is a method to evaluate the test–retest reliability of the diagnostic test [11]. In addition to inter- and intraobserver reliability, it is important to take into account other statistical measures, such as sensitivity, specificity and positive and negative predictive value, when evaluating the accuracy of a diagnostic test [1, 2, 10, 28].

The aim of this study is to assess the inter- and intraobserver reliability and other diagnostic parameters of non-weight-bearing foot radiographs compared with CT in Lisfranc injuries.

## Materials and methods

To assess the accuracy of the diagnostics of Lisfranc injuries, we analysed all foot and ankle CT and CBCT scans acquired due to acute foot trauma at one university hospital and one regional hospital during the period 1.1.2012–31.12.2016. Intra-articular fractures and avulsion fractures around the TMT joint complex were defined as Lisfranc injury. Patients with extra-articular metatarsal injuries were excluded. In addition to the radiologists’ report, the CT scans were separately evaluated by two experienced foot surgery experts. In the case of disagreement, the final diagnosis was made together.

In total, the data comprised 456 patients with acute foot injuries. The CT scans revealed 202 patients without any signs of injury, 21 patients with distal metatarsal or toe fractures and 233 patients with a bony injury (joint displacement, intra-articular or avulsion fracture) affecting the Lisfranc joint complex. The patients were divided into displaced and nondisplaced injuries with a threshold of 2 mm of displacement according to the previous literature [6]. Therefore, injuries with a fracture displacement or TMT joint dislocation of less than 2 mm were considered to be non-displaced and those with 2 mm or more were considered to be displaced. Altogether, 174 patients had a non-displaced Lisfranc injury and 59 patients had a displaced Lisfranc injury. IBM SPSS 24.0 statistical software was used to randomly select 100 patients for the present (reliability) study: 34 patients without a Lisfranc injury (some had distal foot fractures), 33 patients with a non-displaced Lisfranc injury and 33 patients with a displaced Lisfranc injury. The characteristics of the patients are presented in Table 1.

**Table 1:** Characteristics of the patients

	*n* = 100
Age, mean (SD)	40.9 (18)
Males, *n* (%)	55 (55%)
Right foot, *n* (%)	58 (58%)
Patients with Lisfranc injury	*n* = 66
Trauma mechanism, *n* (%)	
Tumbling or slipping	25 (38)
Traffic collisions	11 (17)
Direct injury	8 (6)
Other	22 (37)

The anonymised primary non-weight-bearing foot radiographs were assessed independently by three senior orthopaedic surgeons (with a minimum of 10 years’ experience) and three orthopaedic surgery residents (from 4 to 6 years’ experience) twice at intervals of three months. All 100 sets of radiographs were performed in antero-posterior, 30° oblique and lateral views. The observers were asked to answer the following questions: “Is there an injury at the Lisfranc joint”; (Yes/No), “If you answered yes, describe the findings” and “Are there any other injuries”; (Yes/No).

The sequence of the sets was randomly mixed for the second observation. Picture archiving and communications system (PACS) software was used to display the radiographs.

### Statistical analysis

Fleiss kappa (*κ*) was used to evaluate the interobserver reliability between all six observers in two different moments. Cohen kappa (*κ*) was used to assess the intraobserver reliability between the same observer in two different moments at an interval of three months. Results were presented according to Landis and Koch criteria: 0.00–0.20, slight agreement; 0.21–0.40, fair; 0.41–0.60, moderate; 0.61–0.80, substantial; and 0.81–1.00, almost perfect [24]. The clinical characteristics of the patients are presented as means with standard deviations (SD), medians with interquartile ranges (IQR), as counts with percentages, or as ranges. Differences between means of continuous variables were compared with Mann–Whitney test. False-positive rate was calculated as false negatives divided with CT-positive cases, and false-negative rate was calculated by dividing the false-positive cases with CT-negative cases. Microsoft Excel (version 16.15) and R (version 3.6.0) statistical software were used to conduct statistical analyses.

## Results

When interobserver reliability of non-weight-bearing radiographs in Lisfranc injury was assessed between 6 observers, the κ coefficient for interobserver reliability resulted in moderate correlation from *κ* = 0.50 (95% CI 0.45–0.55) (first evaluation) to *κ* = 0.58 (95% CI 0.52–0.63) (second evaluation). The evaluation was repeated after three months and the κ coefficient for intraobserver reliability between the two evaluations of individual observers showed substantial correlation of mean *κ* = 0.71 (from 0.64 to 0.85) (Table 2).

**Table 2:** Results of the observers’ two evaluations

	Observer 1		Observer 2		Observer 3		Observer 4		Observer 5		Observer 6	
Sensitivity	83.3	83.3	63.6	69.7	60.6	74.2	74.2	75.8	92.4	89.4	77.3	69.7
Specificity	79.4	76.5	100.0	94.1	94.1	82.4	94.1	88.2	52.9	76.5	85.3	100.0
PPV	88.7	87.3	58.6	95.8	95.2	89.1	96.1	92.6	79.2	88.1	91.1	100.0
NPV	71.1	70.3	100.0	61.5	94.1	62.2	65.3	65.2	78.2	78.8	65.9	63.0
Missed cases	11	11	24	20	26	17	17	15	5	7	15	20
False positive	7	8	0	2	2	6	2	4	16	8	5	0
Subtle												
Sensitivity	72.7	75.8	45.5	57.6	51.5	60.6	66.7	69.7	84.8	78.8	69.7	51.5
Missed	9	8	18	14	16	13	11	10	5	7	10	16
Severe												
Sensitivity	93.9	90.9	81.8	81.8	69.7	87.9	81.8	84.8	100.0	100.0	84.8	87.9
Missed	2	3	6	6	10	4	6	5	0	0	5	4
Cohen's kappa (95% CI)	0.85 (0.74–0.96)		0.68 (0.53–0.82)		0.67 (0.53–0.81)		0.70 (0.56–0.84)		0.71 (0.56–0.86)		0.64 (0.50–0.79)	

*PPV* positive predictive value, *NPV* negative predictive value, *CI* confidence interval

The mean (range) sensitivity of all observers was 76.1% (60.6–92.4) and specificity was 85.3% (52.9–100) (Table 2). The sensitivity of the diagnostics in non-displaced injuries was lower than in displaced injuries (65.4% vs 87.1% *p* = 0.003). The number of missed cases was higher among non-displaced injuries than in displaced injuries (*n* = 11 vs 4 *p* = 0.002). The false-negative rate was 23.9% and the false-positive rate was 14.7%. There were no statistically significant differences between senior orthopaedic surgeons and residents in sensitivity (72.5% vs. 79.8%, *p* = 0.44), specificity (87.7 vs. 82.8%, *p* = 0.92), positive predictive value (85.8% vs. 91.2%, *p* = 0.31) or negative predictive value (76.5% vs. 69.4%, *p* = 0.31).

**Fig. 1:**
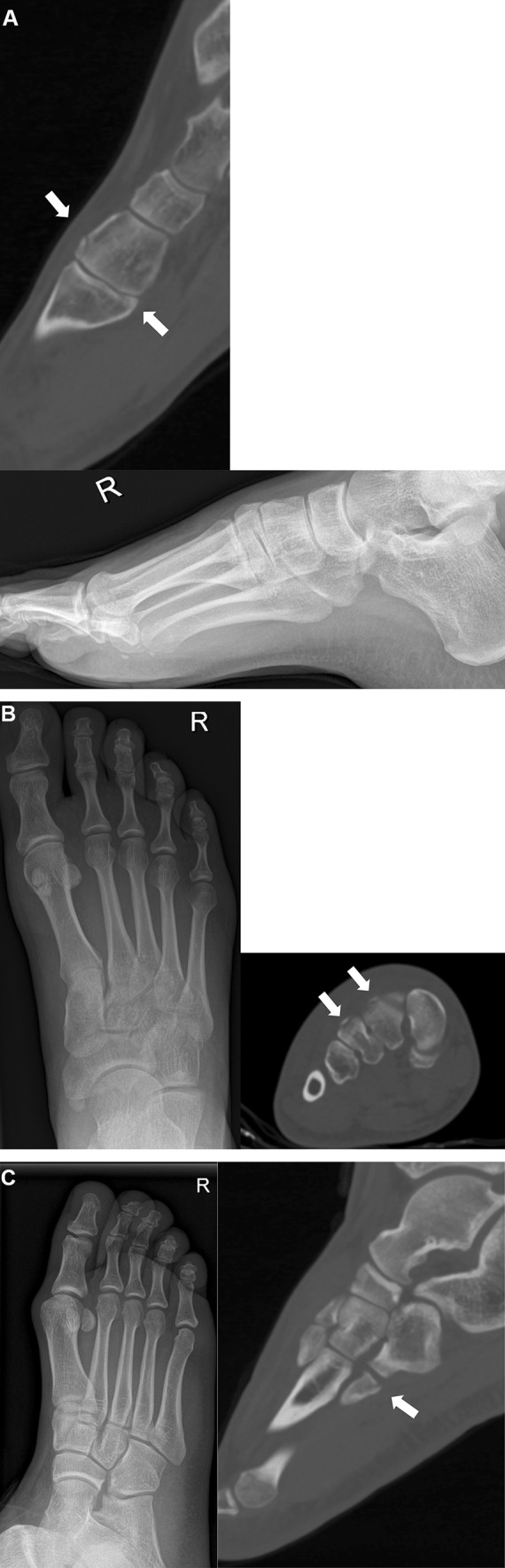
**a**–**c** Radiological findings of the three undisplaced injuries which were missed by all observers. **a** No specific findings with standard radiographs, yet CT revealed fractures of the first metatarsal base and medial cuneiform. **b** No specific findings with standard radiographs, yet avulsion fractures of the second, third and the fourth metatarsal bases were detected in CT. c No specific findings with standard radiographs, yet avulsion fractures of the fourth metatarsal base were detected in CT

**Fig. 2:**
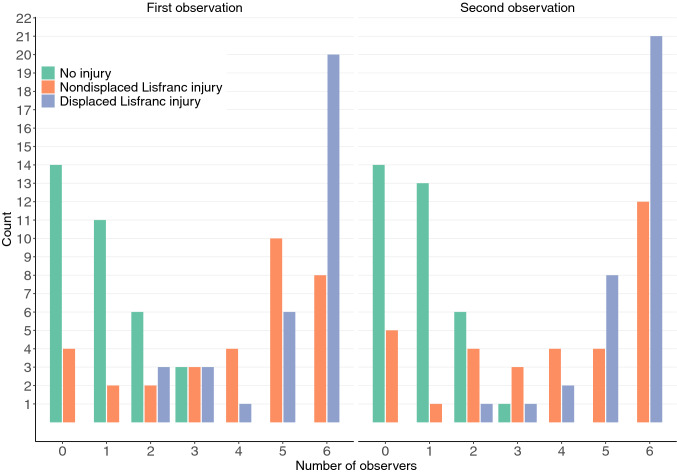
The distribution of the agreement between the observers. Green bars indicate that the non-injured patients were detected with relatively high consensus. Blue (displaced) and orange (nondisplaced) bars represent the agreement between the patients with Lisfranc injury

Consensus between all evaluators was achieved in 38 (38%) cases: 26 cases with an injury and 9 cases without an injury were identified correctly by all evaluators during both evaluations. Three cases with a non-displaced Lisfranc injury were missed by all evaluators (Fig. 1a–c). The agreement was compared with the true positive cases detected by CT (Fig. 2). Results demonstrate that a mild consensus was achieved among most of the non-injured patients, without a significant number of false positives. In the case of injured patients, the consensus was not achieved as precisely, and multiple patients were missed by most of the observers.

## Discussion

The diagnosis of Lisfranc injury based on conventional radiographs had moderate agreement between observers and substantial agreement between the same observer at different time moments. To the best of our knowledge, our study is the first to evaluate the inter- and intraobserver reliability among non-weight-bearing radiographs in the detection of Lisfranc injuries. The main results of our study were that the inter- and intraobserver reliabilities in detecting Lisfranc injuries from non-weight-bearing radiographs depend on the observer, and if the same observer evaluates the same images in different moments, the results will fluctuate. There was some variance in intraobserver reliability among the observers, ranging from substantial agreement to almost perfect. Nondisplaced injuries were significantly more commonly missed than the displaced injuries.

In a previous study by Sherief et al. [48], three radiologists, three orthopaedic surgeons and three physicians evaluated 30 sets of radiographs [48]. The mean sensitivity for Lisfranc injuries was 92% (95% CI 89–95%), and the rate of missed injuries was 19% [48]. They did not report differences between the radiologists, orthopaedic surgeons or physicians. Haapamäki et al. [16] studied the accuracy of the radiological diagnostics of Lisfranc injuries by comparing the findings of 17 conventional radiographs with CT. They presented a sensitivity of 76% and a missed injury rate of 24% for Lisfranc injuries [16]. In addition, Rankine et al. [42] presented a study with 60 non-weight-bearing foot radiographs with 45 CT-positive cases were evaluated by two independent radiologists. They presented a sensitivity of 84.4%, specificity of 53.3% positive predictive value of 84.4% and negative predictive value of 53.3% [42]. In our study with 100 cases, the sensitivity (76%) was comparable to the numbers presented in earlier studies, where the sensitivity has been between 76 and 92% [16, 42, 48]. There were no differences between the senior orthopaedic surgeons and residents in our study, a similar finding to the study of Sherief et al. [48].

Instability of the foot arch, seen as widening of the space between the first and second TMT joints, has been suggested to be the main indication to proceed with operative treatment [3, 40, 46]. Previous studies have criticised the accuracy of non-weight-bearing radiographs in the diagnostics of Lisfranc injuries, since they can only reliably detect severe displacement of the Lisfranc joint and instability is difficult to assess [13, 15, 32, 51]. To correct this flaw, it has been suggested that weight-bearing radiographs are used [3, 7, 9, 15]. However, the problem with weight-bearing radiographs is that the severity of pain usually prevents the patients from reliably bearing weight, and therefore it is impossible to obtain reproducible images [50].

The study by Goiney et al. [14] was the first to describe the benefits of using CT over non-weight-bearing radiography. Since then, the advantages of CT have attracted more interest [26, 38]. The biggest benefit of CT is that small bony displacements, avulsion fragments and fractures are detectable; whereas, they would be missed in non-weight-bearing radiography [26]. To the best of our knowledge, the only study comparing these different imaging modalities in the same sample of Lisfranc injuries was performed by Preidler et al. [38]. They compared the differences between conventional radiography, weight-bearing radiography, CT and MRI with a sample of 49 patients. Their conclusion was that weight-bearing radiographs or MRI do not provide any additional benefit when compared with conventional radiography, and that CT is the most sensitive imaging modality for detecting Lisfranc injuries.

The previous literature provides at least 15 different classification systems for Lisfranc injuries [18, 25, 30, 33, 45, 49]. Injury classifications should be developed as tools to help doctors in decision-making and in choosing the optimal treatment for each patient [4]. Accurately working classifications should also provide estimates of the outcomes after the chosen treatment [4]. In addition, the classifications should have a high inter- and intraobserver reliability to ensure reliability and responsiveness [4]. The inter- and intraobserver reliabilities have been evaluated for the radiograph-based Hardcastle [18] and Myerson [30] classifications for dislocated Lisfranc injuries [27, 52]. Moreover, the inter- and intraobserver reliabilities for these classifications have varied from moderate to excellent [27, 52]. Since most of the previous classifications are based on non-weight-bearing radiographs, we feel it is essential to evaluate the reliability and responsiveness of this imaging modality.

As the use of CT as a diagnostic tool has gained more popularity, the most recently published classifications for Lisfranc injuries have been based on CT imaging [25, 45]. The most recent CT-based classification, the Column Involvement Severity System by Schepers and Rammelt [45], divides Lisfranc injuries according to the columns of the midfoot. The classification represents the affected columns: medial, central and lateral, with the severity of the injury, classified as 0—no joint involved, 1—pure ligamentous with avulsions, 2—simple fracture and 3—comminuted fracture. They suggest that instability is evaluated either by weight-bearing radiographs or stress radiographs under anaesthesia one week after the injury. However, as previously stated, neither of these modalities has been shown to be reliable in detecting the instability [31, 38]. In addition, this classification does not help to choose between nonoperative or operative treatment or to predict the outcome after the chosen treatment.

The strength of our study was the large data sample that included a broad range of Lisfranc injuries. Since the term ‘Lisfranc injury’ is indicative of a wide variety of different injuries in terms of severity, displacement and number of affected joints, it is essential to evaluate the diagnostics with an appropriate study sample [18, 30, 33, 43]. The limitation of our study was that the radiographs were only evaluated by orthopaedic surgeons and orthopaedic surgery residents who are familiar with Lisfranc injuries. However, most of the initial diagnostics occurs in primary healthcare, and patients are then referred to specialized medical care units. Hence, the initial evaluation is often performed by general physicians and it can be assumed that the precision of the diagnostics may be even weaker than the results presented in this work. In addition, the lack of using MRI, weight-bearing CT or weight-bearing radiographs can be considered as a limitation, since some patients with purely ligamentous injuries could be missed.

Since our results show that a significant number of patients would be missed by conventional radiographs, we feel that it is essential to confirm the diagnosis with CT imaging if the clinical suspicion of the injury is high (plantar ecchymosis, pain in active and passive movements or swelling) [9, 44]. Furthermore, there is a need for an accurate injury classification for Lisfranc injuries, based on CT, that would help the clinician with the decision-making and would predict the outcomes after the chosen treatment. Although the classification by Schepers and Rammelt [45] has introduced a novel approach to these injuries, it still requires some further evaluation before it can be used as a tool for choosing the correct treatment for patients.

To conclude, the radiologic diagnosis of a Lisfranc injury based on conventional radiographs has moderate agreement between observers and substantial agreement between the same observer in different time moments. The sensitivity and reliability for detecting Lisfranc injuries with conventional radiographs indicated relatively moderate accuracy. In other words, a substantial number (24%) of injuries are missed if only non-weight-bearing radiographs are used.
